# Optimizing the study planning phase in an investigator-initiated multicenter clinical trial: Mapping the process into a blueprint

**DOI:** 10.1017/cts.2026.10792

**Published:** 2026-07-22

**Authors:** Shannon Hillery, Ryan Majkowski, Esther Woo, Elizabeth Holthouse, Andrew Mould, Lindsay M. Eyzaguirre, Nichol McBee, Karen Lane

**Affiliations:** Johns Hopkins University School of Medicine, Baltimore, MD, USA

**Keywords:** Multicenter clinical trials, clinical trial planning, parallel processing techniques, clinical trials as topic, team science

## Abstract

**Introduction:**

Efficient, precise operationalization of the clinical trial planning phase is critical to timely trial initiation and overall success. Early missteps – imprecise protocols, unclear manuals, delayed training, or incomplete regulatory preparation – create cascading inefficiencies across submissions, data systems, contracting, and site onboarding. Although literature recommends streamlining procedures, mapping the process, and using checklists, there is limited guidance on how to operationalize the planning phase into an executable, time-bound plan.

**Methods:**

Drawing on operational experience from more than 30 multicenter trials and stakeholder interviews, we developed a study planning “blueprint” that maps milestones, roles, tasks, dependencies, and deliverables across a pre-award run up period and planning year (∼15 months). The blueprint prescribes a multi-team structure (Data Analysis and Statistical Reporting; Data Systems Integration; Data Management; Operations and Clinical Affairs; Protocol and sIRB; Financial Operations) to enable parallel work streams, with quarterly milestones aligned to funder performance-based expectations.

**Results:**

The blueprint details quarter-by-quarter deliverables – from pre-award risk assessment to site activation and first enrollment. It emphasizes the centrality of a dedicated project manager, predecessor-successor milestones and tasks, dependency-aware project tools, and routine leadership monitoring to identify and mitigate delays. The model is scalable: smaller teams can consolidate roles and extend timelines while preserving parallelism where feasible.

**Conclusion:**

Formalizing the planning phase with a visual, team-assigned blueprint of parallel work streams may increase operational precision, reduce avoidable delays, and accelerate transition from award to participant enrollment. Future work should quantify milestone- and task-level time and cost metrics to optimize blueprints for diverse trial types.

## Background

Operationalizing a clinical trial efficiently and with precision in its earliest study planning phase is critical for determining its overall trajectory and success. Early, rigorous planning increases the likelihood that subsequent phases will progress smoothly, minimizing operational delays that can impede the timely development of new therapies. While much attention is placed on the start-up and clinical phases, planning phase organization is often misunderstood and therefore the time and workforce needed to get work done is underestimated. This first phase is just as vital to control as subsequent phases – even more so – because every downstream activity depends on the stability, clarity, and accuracy established at the outset. Early missteps in study planning tend to compound over time: an imprecise protocol draft, an ill-defined operations manual, or delays in establishing foundational training modules and regulatory documents can trigger cascading inefficiencies that magnify across regulatory submissions, data capture design, contracting, and site onboarding. These considerations highlight the essential need for a robust trial planning process with a clearly defined workflow structure and a strong infrastructure to ensure that the proposed work is both feasible and maximally impactful.

Much work has been done to mitigate administrative delays by modernizing site selection questionnaires [1], master agreements and other trial tools, but procedural and structural roadblocks, such as cross-communication gaps, circular mismatch errors, and redundancies, continue to delay investigator-initiated trial planning, emphasizing the need for mapping study planning tasks in detail, that is, identifying and visually organizing the study planning phase into milestones, tasks, roles, dependencies and deliverables required to transition into implementation.

Dilts and Sandler classified barriers to opening clinical trials as three types: procedural, structural, and infrastructural [2]. Prime examples of procedural barriers are the numbers of sequential approvals, departmental and review committees, prolonged, unnecessary back-and-forth contract negotiations, and signatories necessary for subcontracts, protocols, and consent forms. Structural barriers are more complex and include circular mismatch errors, for example, a contract officer requiring information that cannot be collected until a data trust review is completed but is being held up without information from the contracting office. And finally, there are infrastructural barriers, such as not enough staff to support coordinating center activities. Dilts and Sandler mapped more than 110 planning steps to open an oncology trial, noting that approximately 50% of the steps added no value, suggesting a need to truncate trial planning steps and redundant reviews.

In discussing the challenges to open clinical trials, Vose et al. suggest mapping institutional processes to eliminate unnecessary duplicative steps while paying particular attention to those steps that are necessary for full compliance with applicable mandatory standards, and to establish clear timelines, milestones, and responsible roles to meet benchmarks of expected productivity [3]. They recommend mapping to optimize timelines and operational efficiencies.

The literature found supports the need for an engaged project manager (PM) to run a clinical trial [2,4–6] and the need for a planning blueprint that includes procedural streamlining, stakeholder alignment, and task mapping, as cited above; but the literature is extremely sparse on how to actually operationalize the trial planning phase. Existing literature includes task checklists and how they may track and impact regulatory and site start-up performance [6,7]. When planning phase operations are analyzed and reported, however, there is a critical gap between identifying the need for trial planning improvement and explicit approaches on how to manage it. This is further compounded by trial planners who are unaware of the barriers ahead and the ways in which to circumnavigate them.

The National Institutes of Health (NIH) recognizes the value of a planning period for the development of essential elements of a clinical trial as evidenced by the R34 and UG3/UH3 grant mechanisms that support principal investigators (PIs) in developing their clinical trial protocols [8]. Despite this mechanism’s support of establishing a research team and developing tools for data management and oversight, the application guidelines lack details on how to transition from notice of award (NOA) to enrollment activation.

This paper introduces a full description of trial planning by visually organizing milestones, timelines, and tasks by roles and in a chronological order over four or five quarters in what we call a planning blueprint. By optimizing the earliest phase – where inefficiencies often originate – we propose that using a blueprint could increase operational precision, reduce the risk of avoidable delays, and accelerate the time to opening a trial to its clinical phase.

## How the study planning blueprint was developed

Based on our operational experience as an academic clinical research organization conducting more than 30 multicenter trials over the last few decades, our blueprint reflects an experience-informed design. This experience led to the development of a blueprint that details team organization, quarterly milestone goals, and parallel activities to meet funding milestone goals. This plan is divided into five calendar quarters, including a pre-NOA period, over approximately 15 months, spanning the time from anticipation of an award through site activation to the clinical phase. The tasks were mapped and assigned to the individuals responsible in the defined roles utilized in the studies.

**Figure 1.**
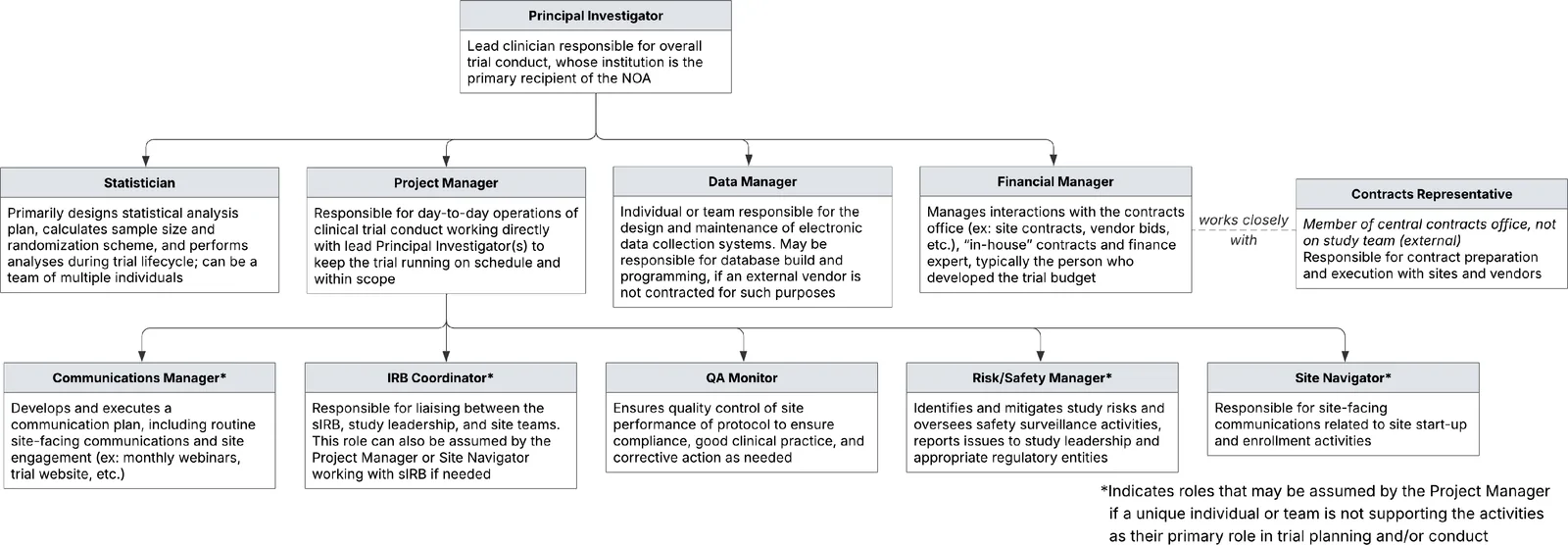
Study planning role definitions. Investigator-initiated multicenter trial coordinating center composition to carry out the planning phase six-team blueprint; however, a smaller team can accomplish the planning phase within a reasonable time. A smaller core team might consist of the principal investigator and a project manager working with a QA monitor, data manager, and financial manager. Asterisks denote roles that are assumed by the Project Manager or another team member if a dedicated person or team is not available. At a minimum, teams should include a QA monitor, data manager, financial manager, and a statistician.

The blueprint is scaled to larger, NIH-funded, investigator-initiated multicenter trials with full clinical coordinating center (CCC) and data coordinating center (DCC) infrastructure and roles, as described in Figure 1. Therefore, research teams without the resources of a CCC or DCC will need to consolidate responsibilities among fewer staff and extend the study planning timeline, since they will have less capacity to perform tasks in parallel, keeping in mind that multicenter trials with limited personnel must still complete the same (or largely similar) set of study-planning tasks regardless of the number of sites or the complexity of the protocol. Alternatively, investigative teams can assign multiple roles to a single individual, enabling that person to work on several tasks concurrently. A small team of core members, each performing multiple roles, can accomplish the planning phase within a reasonable time, especially when using a blueprint to increase operational precision and reduce the risk of avoidable delays. Such a core team might consist of the principal investigator and a PM working with a quality assurance (QA) monitor, data manager, and financial manager. Regardless of trial size, during the study planning phase, the leadership team must scale the planning phase timeline in consideration of the team resources and personnel available.

The effort to formally organize the study-planning phase into a blueprint began in 2021 as a fellowship project that provided a pre-doctoral researcher (author SH) with hands-on clinical research experience. That work produced a task map with timelines, informed by interviews with stakeholder groups experienced in study planning, including but not limited to (1) statisticians; (2) data managers (both programmers and system administrators); (3) research faculty members specializing in multicenter trial management; (4) sIRB directors and experts; and (5) institutional contracts and legal representatives. We distributed the task lists to trial managers for use and feedback from 2021 to 2022. We utilized this feedback to define task sequences and dependencies needed to achieve individual milestones and then grouped the individual milestones into a blueprint concentrating on time-bound quarterly milestones. Next, the tasks and milestones were organized into six teams identical to the stakeholder groups mentioned above. In 2022, we piloted the six-team structure in a large multicenter trial, requiring more than 100 sites and four individual coordinating centers, and were able to meet funder-mandated planning milestones in early 2023. This design enhanced coordination of work, kept tasks progressing in parallel, and aligned with funder-defined milestones.

### Start with the conditions and expectations of the research plan and award terms

Trial planning for an investigator-initiated study begins with the grant application, its accompanying budget justification, and the NOA, which outlines the terms, conditions, and expectations for the research team (Table 1). These foundational documents serve as the basis for organizing the trial planning process into distinct categories and, ideally, into working teams, each responsible for milestones that must be completed before a trial can launch. Once the blueprint is aligned with the award deliverables, the teams become in unison with the NOA expectations, whether the actual NOA or any of its sections are shared with the teams.

**Table 1.** Organizing the Trial Planning Tasks into Distinct Categories and Teams. The grant application, budget justification, and the Notice of Award (NOA) documents provide information for planning phase execution. Deliverables named in these source documents are then assigned to six planning teams Table 1 long description.

Source of information	Document sections providing information for trial planning execution					
Grant Application Section (Document 1)	Statistical Design and Power	Milestones/Timelines Communications Plan	SOA DMS Plan	RMPs (multiple)	Protocol (Specific Aims, Research Strategy, SOA) Human Subjects Protections IRB Activities	Budget Justification Key Personnel Organization Chart
Budget Justification (Document 2)			Enrollment Center Fees	Regulatory Start-Up Fees Enrollment Center Fees	Standard of Care Analysis	Personnel Effort Site Fixed and Per Patient Costs Vendors and Supplies
NOA (Document 3)		Funder-Mandated Milestones Project Period Restrictions or Special Conditions		Regulatory and Compliance		Award Amount Budget Limitations
Assigned to 6 Key Planning Teams	Team 1	Team 2	Team 3	Team 4	Team 5	Team 6
	Data Analysis & Statistical Reporting	Data Systems Integration	Data Management	Operations & Clinical Affairs	Protocol & sIRB	Financial Operations

DMS= Data Management and Sharing; NOA= Notice of Award; IRB = Institutional Review Board; RMP = Risk Management Plan; sIRB = Single Institutional Review Board; SOA= Schedule of Activities.

### Convert the expectations into team assignments

The goal is to implement a multi-team structure to enable parallel progress – a critical factor in accelerating the trial timeline to meet NOA-defined milestones (Table 2). These study planning teams are temporary and distinct from the standard multicenter trial committee structures (i.e., steering committee, executive committee, treatment effects monitoring committee, performance advisory committee, publications committee). Each team should have a designated lead and a backup lead and maintain accountability through regular meetings and quarterly milestone tracking. If multiple CCC/DCC institutions are involved, it will be essential to represent each institution across the teams to ensure coordinated efforts.

**Table 2.** Composition of Six Planning Teams Cross-Referenced to Source Documents Each Team Will Need. Teams are composed of personnel available in Figure 1. Specific documents are cross referenced and distributed among team members to ensure consistent understanding of the protocol, regulatory requirements, and operational procedures in preparation for delivering the milestones listed in Table 3 Table 2 long description.

	Team 1	Team 2	Team 3	Team 4	Team 5	Team 6
	Data Analysis & Statistical Reporting	Data Systems Integration	Data Management	Operations & Clinical Affairs	Protocol & sIRB	Financial Operations
**Group Leader**	Statistician	Project Manager	Data Manager	Project Manager	Principal Investigator	Financial Manager
**Back-up Leader**	Principal Investigator	Data Manager	Project Manager	Principal Investigator	Project Manager	Project Manager
**Suggested Composition**	Statistician(s)	Data Manager(s)	Project Manager	Site Navigator(s)	IRB Coordinator	Principal Investigator
	Data Manager(s)	Risk/Safety Manager	Data Manager(s)	QA Monitor(s)	Statistician	Contracts Representative
	QA Monitor(s)	Financial Manager	Statistician	IRB Coordinator	Data Manager	
		Communications Manager	QA Monitor(s)	Risk/Safety Manager	QA Monitor(s)	
				Data Manager	Financial Manager	
				Communications Manager		
**Cross Reference to Grant/NOA Sections and Additional Documents**	Statistical Design and Power	Protocol	Protocol	Specific Aims	Specific Aims	Organization Chart
	Protocol	Target Enrollment Tables	Target Enrollment Tables	Protocol	Research Strategy	Budget Justification
	Target Enrollment Tables	Communications Plan	SOA	Organization Chart	Protocol	Task Allocation
		Organization Chart	DMS Plan	SOA	SOA	FOA
		Budget		RMP	IRB Required Documents	NOA
		Task Allocation				
		NOA				

DMS = Data Management and Sharing; FOA = Funding Opportunity Announcement; IRB = Institutional Review Board; NOA = Notice of Award; QA = Quality Assessment; RMP = Risk Management Plan; sIRB = Single Institutional Review Board; SOA = Schedule of Activities.

**Table 3.** Quarterly planning blueprint. The six teams in Table 2 are joined to Table 3 to form the planning blueprint, organized into quarterly periods as parallel milestones to achieve during the planning phase. Milestones can be deconstructed into task checklists (Figure 2) by the PM to visualize progress toward milestones. It is recognized that trial planning timelines and milestones may vary across studies; each investigative team is encouraged to customize its planning strategy by assigning teams or individuals and establishing quarterly milestones that reflect the unique requirements and timeline of the trial Table 3 long description.

	Team 1	Team 2	Team 3	Team 4	Team 5	Team 6
	Data Analysis & Statistical Reporting	Data Systems Integration	Data Management	Operations & Clinical Affairs	Protocol & sIRB	Financial Operations
Pre-NOA (Run-Up Period) Milestones	Randomization Scheme Trial Plans Drafted	Risk Assessment	Data Capture Designs Drafted	Site Assessment Survey Readied	Protocol and sIRB Materials in Specified Format	Executive Committee Selected Subcontract/SOW Templates Vendor Bid Requests
	*PM Develops Trial-Specific Detailed Task Lists*					
Quarter 1 Milestones	MOP Skeleton	eTMF Built	EDC/PRO Built	Website and Site Materials	Submit protocol/ICF documents to sIRB Site Selection	Subcontracts Prepared
	*PM Develops Trial-Specific Detailed Task Lists*					
	**Funding agency performance-based phased-award Milestone: Submit finalized protocol and ICF documents**					
Quarter 2 Milestones	DSMB Meeting DSMB approved DSMP as MOP Chapter	Dashboards and Metric collection tools	EDC/PRO UAT Draft EDC/PRO Training Module	Site Start-Up Ready Training Plan as MOP Chapter	sIRB Approval Complete Reliance Agreements Site Training Modules	Partially Execute Subcontracts Execute Vendor Subcontracts
	*PM Develops Trial-Specific Detailed Task Lists*					
	**Funding agency performance-based phased-award Milestone: DSMB review & approval: protocol, ICF and DSMP**					
Quarter 3 Milestones	Randomization Scheme Programmed	Trial Website Launch Communications Plan	EDC/PRO Systems Live EDC/PRO Training	Activate 25% Sites IP Distribution	Monitoring Plan as MOP Chapter	25% Subcontracts Executed
	*PM Develops Trial-Specific Detailed Task Lists*					
	**Funding agency performance-based phased-award Milestone: 25% sites activated**					
Quarter 4 Milestones	SAP as MOP Chapter	IP, RMP, and QA Monitoring as MOP Chapters	Data Management Plan as MOP Chapter	Site Start-Up Program Complete	MOP Distributed	Subcontracts Executed
	*PM Develops Trial-Specific Detailed Task Lists*					
	**Funding agency performance-based phased-award Milestone: 100% sites activated, Enrollment of 1st participant**					

DSMB = Data Safety Monitoring Board; DSMP = Data Safety Monitoring Plan; EDC = Electronic Data Capture; eTMF = Electronic Trial Master File; ICF = Informed Consent Form; IP = Investigational Product; MOP = Manual of Procedures; NOA = Notice of Award; QA = Quality Assessment; PM = Project Manager; PRO = Patient-Reported Outcomes; RMP = Risk Management Plan; SAP = Statistical Analysis Plan; sIRB = Single Institutional Review Board; SOW = Statement of Work; UAT = User Acceptance Testing.

The trial PI and lead PM should work together as the driving force of team organization. Do not underestimate the manpower that will be needed to develop tracking and communication systems. The six-team model includes three data teams to ensure parallel delivery of the Statistical Analysis Plan (SAP), risk assessment plans, and data capture systems. The blueprint includes six planning teams:

1. **Data Analysis & Statistical Reporting Team** – led by the lead statistician and composed of statisticians and data management center leaders. Team 1 defines and documents the trial’s statistical framework.
2. **Data Systems Integration Team** – led by the PM and composed of data managers, data risk/safety manager, and a communications representative, if available. Team 2 focuses on risk-mitigation and integrating the Electronic Trial Master File (eTMF) and metric dashboard with the Electronic Data Capture (EDC) and Patient Reported Outcomes (PRO) systems. This team also develops a trial website, if applicable.
3. **Data Management Team** – led by the lead data manager and composed of the PM, data managers, statisticians, and QA monitors. Team 3 builds and manages the trial participant data capture systems (EDC and PRO) and all related functions.
4. **Operations & Clinical Affairs Team** – led by the PM and composed of CCC leaders and risk managers. Team 4 is responsible for establishing and governing trial oversight and site-facing communications, including training.
5. **Protocol & Single Institutional Review Board (sIRB) Team** – led by the PI and composed of IRB coordinators and representatives from each of the other teams. Team 5 ensures IRB compliance in the trial and participating sites.
6. **Financial Operations Team** – led by the financial manager and composed of those handling contracts and subcontract budgets. Team 6 oversees the trial’s budget and effort allocation.

The teams may be merged into fewer teams if necessary, or milestones may be moved from one team to another. Or for example, a *Regulatory & Compliance Team* could be formed as a 7^th^ team, should a trial require an investigational new drug or device application, depending on legal or regulatory complexity or regulatory goals. The number of teams may vary depending on trial scope, personnel resources, and needs. What is essential is the parallel structure working concurrently toward quarterly milestones to ensure timely trial initiation and that all the milestones are assigned to a team or person(s) early.

### Benchmark quarterly milestones in parallel and in the correct sequence

Table 3 is the blueprint structured as a pre-NOA run-up period then a planning year, divided into quarterly periods. The run-up period adds two to three months to the timeline; however, effort allocation and task assignments during this period should be kept under control, as this period is usually not included in the initial year funds. The advantage of the run-up period is to have a plan in place early, so that once funding comes through, teams hit the ground running and waste less time.

### Funder mandated performance-based phased-award milestones

In NIH-funded trials, a Target Accrual Plan is often required, and other funding sources may have similar trial reporting requirements. These requirements are an important part of any planning blueprint. Table 3 lists the usual agency-imposed milestones (how many sites and participants and when).

### Quarterly milestone task organization and team coordination

Milestones can be broken down into individual tasks and organized into sequenced, actionable steps for inexperienced teams. Table 3 indicates where sequential task details can be added to reach a milestone. Specifying tasks as a checklist can be critical for inexperienced teams, serving as a weekly report to the leadership and executive committee. During the planning phase, leadership is usually composed of the study chair (the PI), the PM, and the executive committee, all working with the six planning phase teams. Weekly check-in meetings enforce timeline adherence, confirm progress, and supply strategic time management. These meetings can also address potential delays early on and allow for timeline adjustments as needed.

Implementing a complementary reporting structure with a central platform for tracking tasks, deadlines, document versions, and approvals promotes consistency and clarity across teams, making it easier for leadership to digest and act on information and measure progress against timelines. It is important to recognize that many planning activities will occur naturally in parallel, whereas other activities will be interdependent, that is, completion of one task will enable, inform, or delay the initiation of another, whether within the same team’s milestone or across different teams and milestones. Developing a tracking system or platform to monitor checklist assignments, durations, and completion status throughout the study planning process will further enhance performance and allow analyses for future improvements and efficiencies.

### Developing task details

The blueprint serves as the project plan to help trial investigators organize trials in a way that achieves maximum parallel productivity with minimum wasted effort and expense. The PM, as the individual heading up the operation, is the intended primary owner of the blueprint, as delegated by the principal investigator(s). The blueprint is shared as a word document with most stakeholders, but the PM can deconstruct each team’s tasks and milestones into checklists, spreadsheets or more sophisticated, dependency-aware project management tools, as needed. The PM is responsible for adding tasks and dependencies that are specific to a given study and to the detail needed, depending on the experience of the team. Once tasks, teams, and milestones are distributed, edits, adjustments and revisions from the teams will be inevitable and should be acted upon jointly by the PM and the executive committee.

**Figure 2.**
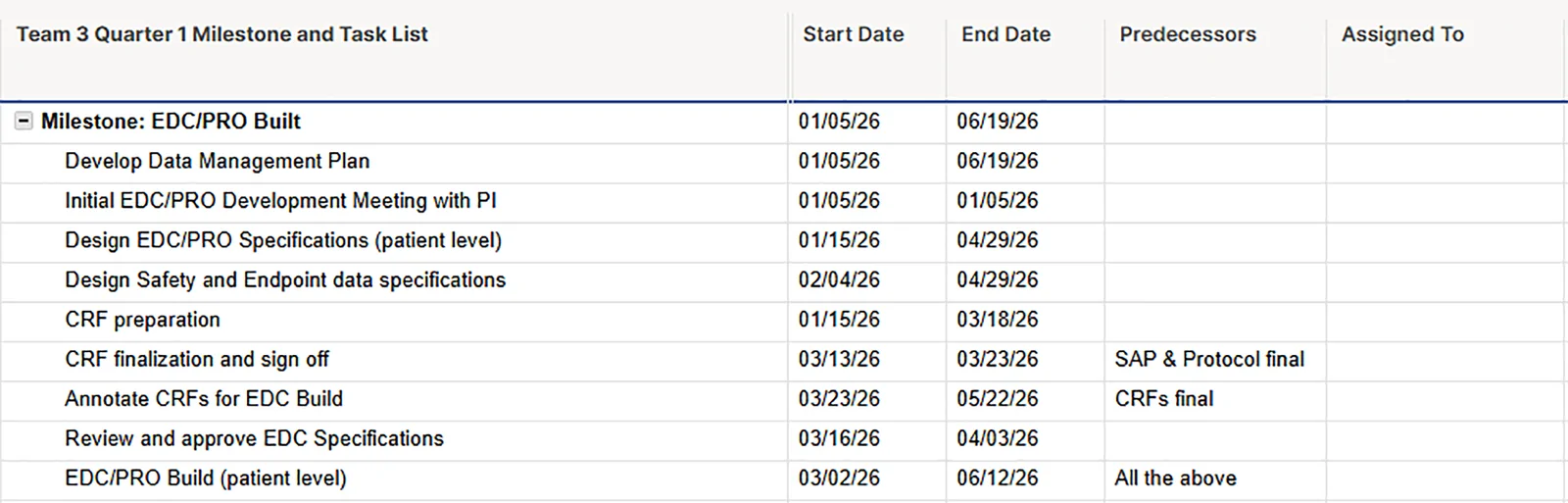
Illustration of how a milestone is broken down into *Individual* tasks. As an example, using a dependency-aware project management tool, the Team 3, Quarter 1 milestone is deconstructed by the PM or assigned Team Leader into a checklist of tasks to meet the quarterly milestone, specifying due dates, predecessor tasks, and responsible individuals.

For each milestone, planning tasks can be broken down into sequential, actionable steps (Figure 2), as follows:

- **Task Description** – A summary of the task to be delivered, such as a document or an event.
- **Start Date** – The scheduled start date is based on the trial timeline. Each goal should be set for the earliest date possible. Date calculations should be sequential to finish dates for prerequisite tasks; the task at hand may shift if preceding tasks are delayed.
- **End Date** – The expected date of task completion to meet the quarterly milestone. Delays here can impact downstream milestones; therefore, negotiate for the earliest possible end date.
- **Predecessor(s)** – Any immediate predecessor task that must be completed before initiating a current task.
- **Assigned To** – The team or individual(s) responsible for executing the task.

### Getting a head start

Strike early following a favorable funding signal. Federal awards permit pre-award costs up to 90 days prior to the date on the NOA (at the grantee’s risk without prior approval, of course) for most grants, but not all. Other agencies or specific funders may be stricter [9]. The PI begins developing a trial task allocation plan to outline the responsibilities of each team. This is a pivotal precursor to assigning team membership. The PM also collaborates with the PI to negotiate the most strategic start date for the award. Other early activities include distributing preliminary trial plans and completing a risk assessment in preparation for the Risk Management Plan (RMP) draft. Initial data capture designs and site assessment strategies can be reviewed by the executive committee, along with the protocol draft, in anticipation of an sIRB submission. Concurrently, the finance team can begin developing subcontract and Statement of Work (SOW) templates and initiate vendor bid requests. Upon confirmation of funding, the budget justification can be formally assigned to the Operations & Clinical Affairs and Financial Operations teams to begin administrative preparations. These initiative-taking steps can accelerate trial readiness and implementation.

During the pre-award period, planning teams can be formed, weekly meetings scheduled on calendars, and the PM and PI can draft the trial planning milestones (see **Convert the Expectations into Team Assignments** above). Together, they prepare and submit whatever immediate documentation the award requires. The PI and PM meet with the executive committee to begin allocating team leaders and members (see Table 2). At a minimum, the planning phase should include a QA monitor, data manager, financial manager, and a statistician, if full team rosters cannot be supported.

#### Pre-award

By the end of a pre-award period, several preliminary tasks should be completed to ensure readiness for the planned year. These include:

- Circulating a draft version of the RMP and randomization scheme
- Translating the grant protocol and SAP into a case report form outline
- Finalization of a site assessment survey, ready for deployment
- Transference of the protocol into a standard sIRB submission-ready format
- Selection of the Executive Committee
- Drafting subcontract and SOW templates
- Conversion of the Budget Justification into an allocation worktable
- Interview vendors for selection

These tasks position the study for a smooth transition into the award phase and a more efficient implementation. For trials that do not have pre-award resources, the PI should begin working on these items as feasible, understanding that remaining preliminary tasks will be pushed into quarter 1 and potentially extend the planned one-year timeline.

### The planning year

#### Quarter 1

Upon receipt of the NOA, full award-driven activities can commence. The teams begin weekly meetings, with the first order of business completing and submitting the sequences of tasks required for each Q1 milestone to be delivered. This includes finalizing budget justification allocations and developing a timeline for new hires, as appropriate. The PM will work with human resource personnel and administrative staff to post-requisitions, using funding documentation as justification.

**Quarter 1 Milestones:** By the end of Award Period Quarter 1, the team(s) will complete the following tasks:

- **Team 1:** Draft the skeleton of the Manual of Procedures (MOP)
- **Team 2:** Build the eTMF
- **Team 3:** Build the EDC and Patient-Reported Outcomes (PRO) systems
- **Team 4:** Develop the trial website and prepare site materials
- **Team 5:** Submit for sIRB approval the study protocol and informed consent form (ICF); Vet and select participating sites (not yet initiated)
- **Team 6:** Prepare subcontracts for selected sites and vendors
- **Funding-Mandated Milestone:** Finalized protocol and ICF documents

#### Quarter 2

All critical preparations for site onboarding should be completed during this quarter, positioning the trial to be “Site Start-Up Ready” at the end of Quarter 2. This readiness enables enrollment centers to begin their start-up activities and satisfies a common funder’s expectation to have a small percentage of sites open to enrollment sometime between months 9 and 12. Specific factors that delay site activation relate to regulatory approval backlogs, training module development put on the back burner and not available to sites when needed, subcontracts not ready, and missing documentation requiring backtracking for correction [10]. A biphasic approach (first finalize all site materials, *then* begin site onboarding) ensures that sites are equipped with all necessary resources for activation and operations, thereby minimizing delays and enhancing overall efficiency.

**Quarter 2 Milestones:** By the end of Award Period Quarter 2, the team(s) will complete the following tasks:

- **Team 1:** Conduct the first Data Safety Monitoring Board (DSMB) meeting and file approvals
- **Team 2:** Develop dashboards and metric collection tools
- **Team 3:** Complete User Acceptance Testing (UAT) of the EDC and Patient-Reported Outcomes (PRO) systems and draft training module
- **Team 4:** Finalize site materials and training modules
- **Team 5:** Acquire DSMB and the sIRB approvals of the protocol and ICF; complete site reliance agreements, add the DSMP to the MOP, and prepare protocol training modules
- **Team 6:** Partially execute subcontracts; execute vendor contracts
- **Funding-Mandated Milestone:** DSMB review & approval of the protocol, ICF and DSMP

#### Quarter 3

Once site start-up ensues in Quarter 3, broader trial planning moves forward in parallel. These concurrent efforts focus on tasks not directly tied to beginning site onboarding, meaning all site start-up materials are done and in the hands of sites at this time. These include finalizing the study website, training site teams on the EDC system, and finalizing investigational product (IP) shipment logistics for when sites activate. Such tasks must be completed prior to beginning Quarter 4, in service of meeting the common funding agency’s performance-based milestone of activating 25% sites at the culmination of Quarter 3.

**Quarter 3 Milestones:** By the end of Award Period Quarter 3, the team(s) will complete the following tasks:

- **Team 1:** Finalize and program the randomization scheme
- **Team 2:** Launch the trial website and complete the Communications Plan
- **Team 3:** EDC and PRO systems live; complete training on EDC and PRO systems
- **Team 4:** Activate 25% of selected sites with at least one site with enrollment; ensure IP is delivered to sites
- **Team 5:** Add the Monitoring Plan to the MOP
- **Team 6:** Fully execute 25% of subcontracts; deliver lab/pharmacy materials to sites
- **Funding-Mandated Milestone:** 25% of sites activated

#### Quarter 4

As site start-up continues, study planning activities end. Remaining activities include finalization of the SAP and other documents that describe the procedures used by the centers as a MOP. The blueprint presented assumes that all policies and procedures developed are eligible topics for a trial MOP; however, the actual contents are decided by the leadership group before development. This parallel workflow ensures that all components of the trial are advancing in synchrony, supporting a smooth transition into full trial implementation.

**Quarter 4 Milestones:** By the end of Award Period Quarter 4, the team(s) will complete the following tasks:

- **Team 1:** Add the Statistical Analysis Plan to the MOP
- **Team 2:** Incorporate IP, RMP, and QA Monitoring into the MOP
- **Team 3:** Add the Data Management Plan to the MOP
- **Team 4:** Complete site start-up with 100% of sites activated
- **Team 5:** Distribute the finalized MOP to all trial stakeholders
- **Team 6:** Ensure all subcontracts are fully executed
- **Funding-Mandated Milestone:** 100% sites activated, Enrollment of 1st participant

## Discussion

Effective planning lays out the groundwork for a streamlined trial, enabling smoother transitions into start-up and enrollment and accelerating the delivery of results that impact patient care. For example, completing protocol-aligned CRFs and SAPs during the planning phase minimizes the need for mid-start-up amendments and database rework, thereby preventing delays to site activation. Similarly, early definition of contracting and regulatory pathways allows site initiation activities to proceed without interruption once subcontracts are issued, avoiding delays by unresolved dependencies. Upfront commitment to careful completion of budgets, plans and materials translates directly into smoother execution during trial start-up and conduct. When the initial phase is tightly organized, with well-structured teams, clear milestones, and transparent communication pathways, it acts as a force multiplier that improves the pace and quality of all later phases. The planning phase sets the parameters for feasibility, resourcing, timelines, and regulatory strategies. It is also the time when interdependencies – such as those between protocol finalization, risk, and data management planning, DSMB preparation, and sIRB submissions – must be recognized, sequenced, and strategically coordinated. Without rigor at this stage, teams often find themselves reworking tasks, correcting preventable errors, or waiting on prerequisites that could have been completed earlier. Thus, while subsequent phases may appear more visible or operationally intensive, the discipline applied during the first phase is what prevents bottlenecks, reduces circular mismatch errors, and enables true parallel processing across teams. In this sense, the early phase is not merely preparatory but foundational: it is the architectural stage in which the blueprint is set, the pathways are mapped and aligned, and the trial’s entire trajectory is shaped. Getting this phase right increases the probability that the more resource‑intensive, time‑sensitive phases that follow can proceed efficiently, without delays or circular work.

In our experience launching multiple multicenter trials each year over multiple decades, this early phase is fraught with pitfalls that are frequently overlooked or not anticipated (Table 4). These include protocol development errors and approval redundancies and draft subcontracts or training modules that are still evolving when downstream teams need them. Other common early-phase pitfalls are the lack of alignment between DSMB, FDA, and sIRB approvals and mistimed creation of study infrastructure (such as behind-schedule development of site and participant materials, EDC systems, trial management platforms, and websites). As Dilts and Sandler describe, redundances, misalignments and late deliverables are “invisible barriers” that can significantly delay progress if not proactively addressed with a knowledge of the work ahead, the details of each task and the dependencies of one task on another known, and with a physical blueprint to help every team member meet these challenges [2]. Without an organizational blueprint with milestones and delivery dates, resources will be misallocated, leading to extended planning timelines and delayed trial implementation [11,12]. To overcome these obstacles, the trial planning phase is best guided by a clearly defined and strategically organized plan built upon a comprehensive understanding of the complex, interdependent relationships among stakeholders, approvals, and milestones to avoid backtracking and inefficiencies. The blueprint can be deconstructed and checklists automated using software that supports formal predecessor–successor relationships and common dependency types, such as Microsoft Project Professional, Smartsheet, or Monday.com. These tools allow automated propagation of schedule changes and visualization of tasks (critical paths), facilitating timely assessment of how task delays affect downstream milestones. Although spreadsheets or document-based checklists are sufficient for small or loosely coupled tasks, they require manual updates and do not scale well for projects with multiple interdependencies. Dependency-aware project management tools therefore provide a more robust and reliable framework for coordinating activities and monitoring progress toward milestones.

**Table 4.** Common early phase pitfalls and mitigation strategies in clinical trial planning. Trial planning is fraught with common barriers and pitfalls. When pitfalls are recognized, mitigation strategies can improve the pace of overall trial planning Table 4 long description.

Category	Planning phase pitfall	Description	Mitigation strategy
Stakeholder engagement	Taking a “wait and see” attitude or under-committing when the funding signal is strong	Statisticians and other trial planners are not integrated early enough to shape planning and sequencing.	Strike early! Engage stakeholders early enough to shape timelines and milestones regarding start date negotiations and milestone commitments. Identify executive committee and DSMB members and medical monitors to be ready for early reviews of new materials.
Team structure and roles	Lack of early team formation and role clarity	Teams formed too late or without designated leads/backups, causing gaps in accountability and slower decision-making. Vague boundaries between responsibilities.	Designate leaders, backup leaders, and communication pathways early, in anticipation of an award. Do not wait until after the award to decide on work allocations.
Task dependencies	Unrecognized or poorly mapped interdependencies	Teams are delayed by predecessor tasks they did not know they were waiting for, leading to circular mismatch errors and rework.	Map all critical path tasks into a published blueprint or similar visual tool to ensure predecessor-successor clarity.
Regulatory pathways	Late identification of regulatory pathways	For example, missed FDA IND/IDE applications result in missed steps and duplicated work correcting protocols and other site-related documents.	Decide early on what regulatory elements will be required and belong under which team, especially when the PI also functions as a sponsor-investigator.
Protocol development	Unclear or evolving protocol delays to finalization	Protocol elements (endpoints, assessments, visit schedules, etc.) are not finalized early, causing downstream delays in EDC design, IRB submissions, and safety planning, etc.	Establish an early protocol team with inputs from biostatistics, medical monitors, and data designers.
Budget and contract	Slow budgeting and subcontracting processes	Subcontracts, vendor bid packages and task allocations are delayed, slowing site preparation and financial negotiations.	Draft the budget and budget justification templates for subcontractors before the funding arrives and as a predecessor to other work. Anticipate that sponsors may require up to six weeks to prepare subcontracts.
Foundational documents	Delayed development of core planning documents	Early materials, such as risk assessment and RMP, trial plans, and task allocations are not created in time to guide subsequent work.	Draft templates from source documents such as the grant, protocol, and budget justification to strike early in anticipation of an award.
Tracking and communication systems	Lack of centralized tracking and communication systems for accountability	Teams operate in silos without shared tools for tracking, document control, or milestone monitoring, leading to redundant efforts and missed dependencies.	Decide on a unified platform for tracking tasks, deadlines, document versions, and approval of audit trails. Couple systems with escalation pathways.
Data systems and data planning	Premature or misaligned systems development	Inadequate planning for EDC, PRO, patient-facing materials, and systems integration. EDC is built before data forms, visit schedule, or protocol elements are finalized.	Declare parallel timelines and align workflows across platforms for EDC, eTMF, PRO, and safety reporting.
Site-related preparations	Incomplete early site onboarding activities	Time required for reliance agreements, sIRB approvals, and Local Context Questionnaires are underestimated.	Assign early deadlines for finalized site materials, including site assessment surveys, training modules, onboarding materials, and vendor-sourced equipment and supplies.
Parallel processing	Excessive reliance on serial or random workflows	Teams default to sequential or random steps even when parallel work is possible, unnecessarily extending timelines	Insist on team compliance with the planning phase blueprint and prepare onboarding materials preemptively for release to all stakeholders.

DSMB = Data Safety Monitoring Board; EDC = Electronic Data Capture; eTMF = Electronic Trial Master File; IDE = Investigational Device Exception; IND = Investigational New Drug; IRB = Institutional Review Board; PI = principal investigator; PRO = Patient-Reported Outcomes; RMP = Risk Management Plan; sIRB = Single Institutional Review Board.

The principles of optimizing workflows and automating processes – such as mapping workflows, using blueprints, task checklists, and benchmarks to streamline timelines – are well-supported. Morgan et al., as well as others, highlight that parallel processing techniques, widely adopted in fields like computer science and manufacturing, are only beginning to be applied within the life sciences sector [13–15]. Regardless of trial size or resource availability, transitioning from traditional serial workflows to parallel approaches will yield considerable time savings. The task at hand for a PM and PI is to plan how the milestones will happen in parallel, taking into account the steps that must be done sequentially and with the personnel available, in a manner that also avoids circular re-work, and to monitor progress across teams so that no individual planning team falls behind on milestones, triggering a domino effect delaying other teams.

## Limitations

There is no universal blueprint for planning all clinical trials. As with any architectural blueprint, variations are necessary to accommodate differences in trial design, staffing capacity, and trial management platforms. This blueprint reflects a high-effort approach tailored for large-scale trials involving multiple core institutions and substantial budgets. For a research team to implement this blueprint fully, adequate funding and resources are essential to scale personnel appropriately. However, with strategic parallel work sequencing, role delineation, and a focus on time-bound milestones, as presented in this blueprint, smaller-scale coordinating centers can enhance the efficiency and pace of the trial planning phase as well and aim to reduce the time to get to site activation and participant enrollment.

Nonetheless, the greatest challenge to meet benchmarked timelines remains the management of unforeseen disruptions. Even the most meticulously organized and timetabled planning teams can be delayed by staffing changes, regulatory hurdles, IP manufacturing delays, or technological issues. Experience shows that few trials are immune to setbacks. Therefore, it is imperative to embrace all feasible strategies to enhance efficiency and uphold operational quality, even if full implementation of a model such as this is not possible.

Factors may limit the generalizability of this proposed organizational blueprint for trials that are already funded and getting underway. However, as these planning concepts gain traction, future investigators may be better equipped to budget and design trials that facilitate smoother and faster transitions from grant award to first participant enrollment.

## Next steps

While no single data source currently exists to guide trial planning comprehensively, the increasing attention to trial metadata offers promising opportunities. These data can provide evidence-based insights into task sequencing and realistic timelines, supporting a shift from sequential to parallel pathways and structured planning approaches. Further research is needed to quantify the time and cost associated with individual trial planning tasks and to identify optimal strategies for mapping the most efficient, adaptable blueprints tailored to various clinical trial types.

## Conclusion

As an academic research organization implementing multiple multicenter trials annually, we experience firsthand how challenging it is to get clinical trials off the ground in a timely manner, despite our focus on streamlining human tasks and procedures. Our experience has led to a much better understanding of the intricacies of delivering trial materials, how each team’s tasks affect other teams, and how to identify and anticipate critical operational dependencies that will either impede or improve the pace of trial readiness overall.

Our emphasis on clearly articulated structures, and purpose-built infrastructure represents a change in thinking from the traditional model, which as a rule is an underinvestment in early-stage design. Successful trial planning hinges on close collaboration, transparency, and teamwork. Utilizing a shared and well-mapped blueprint during the planning phase may help teams overcome hidden barriers and disentangle complex interdependencies that can impede trial readiness. The literature preceding our work confirms that planning phase checklists are vital to the planning process. We hope, by taking checklists one step further, visualizing work in efficient parallel progression, with timelines, milestones, and sequencing, we are building on checklists and provide new knowledge on how to manage a team through the study planning period.

## Table 1. Long description

A table organizing trial planning tasks into distinct categories and teams. The table has one header, four rows and seven columns. Column headers are Source of information and Document sections providing information for trial planning execution. Row 1: Grant Application Section (Document 1), Statistical Design and Power, Milestones/Timelines, SOA, RMPs (multiple), Protocol (Specific Aims, Research Strategy, SOA), Budget Justification. Row 2: Budget Justification (Document 2), Blank Entry, Blank Entry, Enrollment Center Fees, Regulatory Start-Up Fees and Enrollment Center Fees, Standard of Care Analysis, Personnel Effort Site Fixed and Per Patient Costs and Vendors and Supplies. Row 3: NOA (Document 3), Blank Entry, Funder-Mandated Milestones and Project Period Restrictions or Special Conditions, Blank Entry, Regulatory and Compliance, Blank Entry, Award Amount and Budget Limitations. Row 4: Assigned to 6 Key Planning Teams, Team 1 Data Analysis & Statistical Reporting, Team 2 Data System Integration, Team 3 Data Management, Team 4 Operations & Clinical Affairs, Team 5 Protocol & sIRB, Team 6 Financial Operations. The table details how deliverables named in the grant application, budget justification, and Notice of Award (NOA) documents are assigned to six planning teams. Each team is responsible for specific milestones that must be completed before a trial can launch. The teams align their work with the award deliverables and NOA expectations.

Navigate back to Table 1..

## Table 2. Long description

A table detailing the composition of six planning teams and their cross-reference to source documents. The table is organized into five rows and seven columns, each column represents a different team: Team 1 Data Analysis & Statistical Reporting, Team 2 Data Systems Integration, Team 3 Data Management, Team 4 Operations & Clinical Affairs, Team 5 Protocol & sIRB, and Team 6 Financial Operations. Each row is a designated roles including Group Leader, Back-up Leader, and Suggested Composition, the last row is a Cross Reference to Grant/NOA Sections and Additional Documents. Row-wise data: Row 1: Group Leader, Statistician, Project Manager, Data Manager, Project Manager, Principal Investigator, Financial Manager. Row 2: Back-up Leader, Principal Investigator, Data Manager, Project Manager, Principal Investigator, Project Manager, Project Manager. Row 3: Suggested Composition, Statistician(s) and Data Manager(s) and QA Monitor(s), Data Manager(s) and Risk/Safety Manager and Financial Manager and Communications Manager, Project Manager and Data Manager(s) and Statistician and QA Monitor(s), Site Navigator(s) and QA Monitor(s) and IRB Coordinator and Risk/Safety Manager and Data Manager and Communications Manager, IRB Coordinator and Statistician and Data Manager and QA Monitor(s) and Financial Manager, Principal Investigator and Contracts Representative, Row 4: Cross Reference to Grant/NOA Sections and Additional Documents, Statistical Design and Power and Protocol and Target Enrollment Tables, Protocol and Target Enrollment Tables and Communications Plans and Organization Chart and Budget and Task Allocation and NOA, Protocol and Target Enrollment Tables and SOA and DMS Plan, Specific Aims and Protocol and Organization Chart and SOA and RMP, Specific Aims and Research Strategy and Protocol and SOA and IRB required documents, Organization Chart and Budget Justification and Task Allocation and FOA and NOA.

Navigate back to Table 2..

## Table 3. Long description

A table detailing the milestones for six teams during the trial planning phase, organized into pre-NOA (Run-Up Period) Milestones, Quarter 1, Quarter 2, Quarter 3, and Quarter 4 periods. The teams are; Team 1 Data Analysis & Statistical Reporting, Team 2 Data Systems Integration, Team 3 Data Management, Team 4 Operations & Clinical Affairs, Team 5 Protocol & sIRB, and Team 6 Financial Operations. Each team has specific tasks and milestones to achieve in each quarter. In the pre-NOA period, Team 1 task: Randomization Scheme Trial Plans Drafted and PM Develops Trial-Specific Detailed Task Lists,, Team 2: risk assessment, Team 3: Data Capture Designs Drafted, Team 4: Site Assessment Survey Readied, Team 5: Protocol and sIRB Materials in Specified Format, and Team 6: Executive Committee Selected and Subcontract/SOW Templates and Vendor Bid Requests. Quarter 1 Milestones, Team 1: MOP Skeleton PM Develops Trial-Specific Detailed Task Lists and Funding agency performance-based phased-award Milestone: Submit finalized protocol and ICF documents, Team 2: eTMF Built, Team 3: EDC/PRO Built, Team 4: Website and Site Materials, Team 5: Website and Site Materials Submit protocol/ICF documents to sIRB Site Selection, Team 6 Subcontracts Prepared. Quarter 2 milestones are Team 1: DSMB Meeting DSMB approved DSMP as MOP Chapter and PM Develops Trial-Specific Detailed Task Lists and Funding agency performance-based phased-award Milestone: DSMB review & approval: protocol, ICF and DSMP, Team 2: Dashboards and Metric collection tools, Team 3: EDC/PRO UAT and Draft EDC/PRO Training Module, Team 4 Site Start-Up Ready and Training Plan as MOP Chapter, Team 5: sIRB Approval Complete Reliance Agreements Site Training Modules, Team 6: Partially Execute Subcontracts and Execute Vendor Subcontracts. Quarter 3 milestones, Team 1: Randomization Scheme Programmed PM Develops Trial-Specific Detailed Task Lists and Funding agency performance-based phased-award Milestone: 25% sites activated, Team 2: Trial Website Launch Communications Plan, Team 3: EDC/PRO Systems LiveEDC/PRO Training, Team 4: Activate 25% Sites IP Distribution, Team 5: Monitoring Plan as MOP Chapter, Team 6: 25% Subcontracts Executed. Quarter 4 milestones, Team 1: SAP asMOP Chapter PM Develops Trial-Specific Detailed Task Lists and Funding agency performance-based phased-award Milestone: 100% sites activated, Enrollment of 1st participant, Team 2: IP, RMP, and QA monitoring as MOP chapters, Team 3: data management plan as MOP chapter, Team 4: site start-up program complete, Team 5: MOP distributed, and Team 6 subcontracts executed. The table also includes performance-based phased-award milestones for the funding agency, such as submitting finalized protocol and ICF documents, DSMB review and approval of protocol, ICF, and DSMP, activating 25 percent sites, and activating 100 percent sites and enrolling the first participant.

Navigate back to Table 3..

## Table 4. Long description

A table with 11 rows and 4 columns. The columns are labeled Category, Planning phase pitfall, Description, and Mitigation strategy. The rows are: Stakeholder engagement Team structure and roles, Task dependencies, Regulatory pathways, Protocol, development, Budget and contract, Foundational documents, Tracking and communication systems, Data systems and data planning, Site-related preparations, Parallel processing. Row 1: Category, Stakeholder engagement; Planning phase pitfall, Taking a wait and see attitude or under-committing when the funding signal is strong; Description, Statisticians and other trial planners are not integrated early enough to shape planning and sequencing; Mitigation strategy, Strike early! Engage stakeholders early enough to shape timelines and milestones regarding start date negotiations and milestone commitments. Row 2: Category, Team structure and roles; Planning phase pitfall, Lack of early team formation and role clarity; Description, Teams formed too late or without designated leads/backups, causing gaps in accountability and slower decision-making. Vague boundaries between responsibilities; Mitigation strategy, Designate leaders, backup leaders, and communication pathways early, in anticipation of an award. Do not wait until after the award to decide on work allocations. Row 3: Category, Task dependencies; Planning phase pitfall, Unrecognized or poorly mapped interdependencies; Description, Teams are delayed by predecessor tasks they did not know they were waiting for, leading to circular mismatch errors and rework; Mitigation strategy, Map all critical path tasks into a published blueprint or similar visual tool to ensure predecessor-successor clarity. Row 4: Category, Regulatory pathways; Planning phase pitfall, Late identification of regulatory pathways; Description, For example, missed FDA IND/IDE applications result in missed steps and duplicated work correcting protocols and other site-related documents; Mitigation strategy, Decide early on what regulatory elements will be required and belong under which team, especially when the PI also functions as a sponsor-investigator. Row 5: Category, Protocol development; Planning phase pitfall, Unclear or evolving protocol delays to finalization; Description, Protocol elements (endpoints, assessments, visit schedules, etc.) are not finalized early, causing downstream delays in EDC design, IRB submissions, and safety planning, etc.; Mitigation strategy, Establish an early protocol team with inputs from biostatistics, medical monitors, and data designers. Row 6: Category, Budget and contract; Planning phase pitfall, Slow budgeting and subcontracting processes; Description, Subcontracts, vendor bid packages and task allocations are delayed, slowing site preparation and financial negotiations; Mitigation strategy, Draft the budget and budget justification templates for subcontractors before the funding arrives and as a predecessor to other work. Anticipate that sponsors may require up to six weeks to prepare subcontracts. Row 7: Category, Foundational documents; Planning phase pitfall, Delayed development of core planning documents; Description, Early materials, such as risk assessment and RMP, trial plans, and task allocations are not created in time to guide subsequent work; Mitigation strategy, Draft templates from source documents such as the grant, protocol, and budget justification to strike early in anticipation of an award. Row 8: Category, Tracking and communication systems; Planning phase pitfall, Lack of centralized tracking and communication systems for accountability; Description, Teams operate in silos without shared tools for tracking, document control, or milestone monitoring, leading to redundant efforts and missed dependencies; Mitigation strategy, Decide on a unified platform for tracking tasks, deadlines, document versions, and approval of audit trails. Couple systems with escalation pathways. Row 9: Category, Data systems and data planning; Planning phase pitfall, Premature or misaligned systems development; Description, Inadequate planning for EDC, PRO, patient-facing materials, and systems integration. EDC is built before data forms; visit schedule or protocol elements are finalized; Mitigation strategy, Declare parallel timelines and align workflows across platforms for EDC, eTMF, PRO, and safety reporting. Row 10: Category, Site-related preparations; Planning phase pitfall, Incomplete early site onboarding activities; Description, Time required for reliance agreements, sIRB approvals, and Local Context Questionnaires are underestimated; Mitigation strategy, Assign early deadlines for finalized site materials, including site assessment surveys, training modules, onboarding materials, and vendor-sourced equipment and supplies. Row 11: Category, Parallel processing; Planning phase pitfall, Excessive reliance on serial or random workflows; Description, Teams default to sequential or random steps even when parallel work is possible, unnecessarily extending timelines; Mitigation strategy, Insist on team compliance with the planning phase blueprint and prepare onboarding materials preemptively for release to all stakeholders.

Navigate back to Table 4..
